# Martius flap in recurrent rectovaginal fistula: a video vignette

**DOI:** 10.1007/s00384-025-05004-7

**Published:** 2025-10-16

**Authors:** Yasemin Yildirim, Cigdem Arslan, Onur Bayraktar, Ilknur Erenler Bayraktar

**Affiliations:** 1 Gayrettepe Florence Nightingale Hospital, Istanbul, Turkey; 2 Private Clinic, Istanbul, Turkey; 3 Liv Hospital VadiIstanbul, Istanbul, Turkey

**Keywords:** Anovaginal fistula, Martius flap, Rectovaginal fistula, Colostomy

## Abstract

**Purpose:**

We aimed to demonstrate the Martius flap treatment in a patient who had a recurrent iatrogenic rectovaginal fistula following cystocele repair.

**Method:**

We performed a Martius flap procedure for the reconstruction. A vascularized flap was harvested from the labial fat pad, ensuring preservation of its blood supply via the internal pudendal vessels. The flap was then transposed to the defect site to provide adequate vascular support and promote healing.

**Results:**

The patient initially underwent a primary repair of the rectovaginal fistula, which did not achieve fistula healing. A diverting colostomy was then created. Following referral to our unit, a Martius flap using tissue from the left labium majus was performed. The patient was discharged on postoperative day 2 without complications.

**Conclusion:**

RVF is a debilitating condition that can significantly impair quality of life and often necessitates repeated surgical interventions. We believe that implementing techniques to facilitate the healing process may play a crucial role in optimizing patient outcomes.

**Supplementary Information:**

The online version contains supplementary material available at 10.1007/s00384-025-05004-7.

## Introduction

Rectovaginal fistulas (RVF) can be classified as "low" if they are located below the dentate line, "high" if they are associated with the rectum, and "mid" for those located between these two regions [1]. The terms "anovaginal fistula" and "low rectovaginal fistula" are often used interchangeably. RVF can also be classified as "simple" and "complex." Simple RVF are located between the anal canal and vagina, with a diameter of less than 2.5 cm. The most common causes are obstetric trauma and infection. "Complex" fistulas are located higher between the rectum and vagina, with a larger diameter [2]. They are often caused by complications from radiation, cancer, or pelvic surgical procedures [3]. Treatment of RVF involves a range of interventions that depend on the presenting symptoms, fistula anatomy, the quality of surrounding tissues, and any prior repair attempts.

The Martius flap is a well-established surgical technique used in the reconstruction of urogenital defects, particularly in cases of vesicovaginal and RVF [3]. The technique involves harvesting a vascularized flap from the labial fat pad, ensuring preservation of its blood supply through the internal pudendal vessels. The flap is then transposed to the defect site to provide robust vascular support and enhance healing.

This video presents the Martius flap technique performed on a 39-year-old female patient with a recurrent rectovaginal fistula for the second time. The patient initially developed an iatrogenic fistula six years ago following cystocele repair. She had previously undergone a primary repair for rectovaginal fistula at another institution. Due to persistent fistula and failure of the initial repair, a diverting colostomy was subsequently created, again at the referring center. At the time of the Martius flap procedure performed by our team, the patient already had a functioning diverting colostomy. She was discharged on postoperative day 2 following the Martius flap surgery. During follow-up, the colostomy was reversed at 3 months postoperatively, and no recurrence of the fistula was observed. The postoperative follow-up period has reached 15 months. No recurrence, complications, or incontinence have been observed during this time.

## Supplementary Information

Below is the link to the electronic supplementary material.

Supplementary file1 (MP4 396351 KB)

## Data Availability

The data, video, and other resources used in this study are freely available online. Researchers who need further assistance or clarification on specific analyses are encouraged to reach out via email, and we are happy to offer support upon request.

## Declarations

Ethics approval was obtained in accordance with the ethical standards of the institutional research committee and the 1964 Helsinki Declaration, along with its subsequent amendments or comparable ethical standards.

## References

[1] Lowry AC. Benign anorectal: rectovaginal fistulas. In: Wolf BG, Fleshman JW, Beck DE, Pemberton JH, Wexner SD, eds. The ASCRS Textbook of Colon and Rectal Surgery. New York, NY: Springer; 2007

[2] Lowry AC, Thorson AG, Rothenberger DA, Goldberg SM (1988) Repair of simple rectovaginal fistulas. Influence of previous repairs. Dis Colon Rectum 31:676–678 3168676 10.1007/BF02552581

[3] Ommer A, Herold A, Berg E, Fürst A, Schiedeck T, Sailer M (2012) German S3-Guideline: rectovaginal fistula. Ger Med Sci 10:Doc15. 10.3205/000166 23255878 10.3205/000166 PMC3525883

